# Research trends of ferroptosis and pyroptosis in Parkinson’s disease: a bibliometric analysis

**DOI:** 10.3389/fnmol.2024.1400668

**Published:** 2024-05-16

**Authors:** Zihua Wu, Kexin Zhong, Biao Tang, Sijian Xie

**Affiliations:** ^1^Key Laboratory of Vascular Biology and Translational Medicine, Hunan University of Chinese Medicine, Changsha, China; ^2^People’s Hospital of Ningxiang City, Hunan University of Chinese Medicine, Changsha, China; ^3^National Key Laboratory Cultivation Base of Chinese Medicinal Powder & Innovative Medicinal Jointly Established by Province and Ministry, Hunan University of Chinese Medicine, Changsha, China

**Keywords:** Parkinson’s disease, ferroptosis, pyroptosis, bibliometric analysis, Citespace, VOSviewer

## Abstract

**Objective:**

This study aims to visualize the trends and hotspots in the research of “ferroptosis in PD” and “pyroptosis in PD” through bibliometric analysis from the past to 2024.

**Methods:**

Literature was retrieved from the Web of Science Core Collection (WoSCC) from the past to February 16, 2024, and bibliometric analysis was conducted using Vosviewer and Citespace.

**Results:**

283 and 542 papers were collected in the field of “ferroptosis in PD” and “pyroptosis in PD.” The number of publications in both fields has increased yearly, especially in “ferroptosis in PD,” which will become the focus of PD research. China, the United States and England had extensive exchanges and collaborations in both fields, and more than 60% of the top 10 institutions were from China. In the fields of “ferroptosis in PD” and “pyroptosis in PD,” the University of Melbourne and Nanjing Medical University stood out in terms of publication numbers, citation frequency, and centrality, and the most influential journals were Cell and Nature, respectively. The keyword time zone map showed that molecular mechanisms and neurons were the research hotspots of “ferroptosis in PD” in 2023, while memory and receptor 2 were the research hotspots of “pyroptosis in PD” in 2023, which may predict the future research direction.

**Conclusion:**

This study provides insights into the development, collaborations, research themes, hotspots, and tendencies of “ferroptosis in PD” and “pyroptosis in PD.” Overall situation of these fields is available for researchers to further explore the underlying mechanisms and potential treatments.

## 1 Introduction

Parkinson’s disease (PD) is a common degenerative disease of the central nervous system (CNS) (Huang et al., 2024), which has special pathological changes including the degeneration and loss of dopaminergic (DA) neurons in the substantia nigra (SN), decreased DA content in the striatum, and the formation of Lewy bodies dominated by the aggregation of α-synuclein (α-syn) (Naoi et al., 2024). The symptoms include motor symptoms, and non-motor symptoms (NMS) (Israni et al., 2024), which may precede the onset of motor symptoms by several years and increase the risk of overall disability. Currently, some motor symptoms can only be controlled by anticholinergic drugs (e.g., trihexyphenidyl) and dopaminergic drugs (e.g., levodopa), etc., but cannot prevent the progression or reduce the risk of disability (Siripurapu et al., 2023). Therefore, further mechanism study to find therapeutic breakthroughs is the core issues that need to be urgently addressed.

Cell death is a fundamental biological phenomenon that holds significant importance in the growth, development, and maintenance of tissue homeostasis in organisms. Recently, controlled cell death pathways with unique pathophysiological characteristics have been discovered, including apoptosis, necroptosis, autophagy, pyroptosis and ferroptosis, as well as uncontrolled forms of cell death such as necrosis (Qiu et al., 2023). Among them, ferroptosis and pyroptosis have been widely shown to affect the disease progression of PD by modulating pathological processes such as inflammatory response (Huang and Li, 2024) and oxidative stress (Yao et al., 2024). Moreover, targeting key molecules of multiple cell death modes can effectively alleviate PD symptoms.

Ferroptosis is programmed cell death depending on iron accumulation, and is associated with increased levels of iron and reactive oxygen species (ROS), decreased glutathione (GSH) levels, and decreased glutathione peroxidase 4 (GPX4) in cells. Among them, lipid peroxides (LPO) accumulation is a key component of ferroptosis, which is regulated by multiple oxidative and antioxidant systems (Chen X. et al., 2021). Accumulation of iron in the SN has been broadly correlated with the development of PD. Increased iron content in the SN augments free radical production through the Fenton reaction, leading to neuronal degeneration. In addition, iron accumulation has been shown to promote α-syn aggregation, which occurs synergistically, inducing the formation of Lewy body and degenerative necrosis of DA neurons (Ding et al., 2023). Numerous studies have shown that PD patients have increased levels of free iron and LPO, and decreased levels of GSH. Furthermore, genetic mutations involved in iron metabolism may cause increased intracellular iron input or decreased output, constituting a predisposing factor for the development of PD (Wang et al., 2023).

Pyroptosis is an inflammatory cell death mediated by the gasdermin (GSDM) family, including canonical mechanisms mediated by caspase-1 and non-canonical mechanisms mediated by caspase-4/5/11 (Wu et al., 2024). The activation of caspase-1 or caspase-4/5/11, induced by immune activation, cleaves GSDM and leads to pyroptosis (Zhang et al., 2023). In addition, activated caspase-1 is associated with the formation and release of IL-1β and IL-18. It has been demonstrated that pyroptosis is associated with the pathomechanism of PD. α-Syn, a PD pathology-associated protein, promotes the progression of PD by activating microglia and subsequently inducing neuroinflammation after release by damaged neurons. Pyroptosis can increase inflammatory effects to damage DA neurons by promoting IL-1β secretion and activation of inflammasomes. Activated NLRP3 is detected at sites of DA cell loss in the SN of mouse PD model and cerebrospinal fluid of patients with PD. Moreover, the levels of IL-1β and IL-18 in cerebrospinal fluid and serum samples are significantly higher in PD patients than in control (Zheng et al., 2023).

Bibliometrics is a discipline that uses mathematical and statistical measurement methods to study the quantitative relationships and developmental patterns of literature and related information, which can explore the dynamic characteristics and research hotspots of a particular field (Öztürk et al., 2024). Bibliometric analysis typically utilizes various software and algorithms to present the trend of annual publications, countries/regions, institutions, journals, authors, and co-citations within a domain. Given that bibliometric analyses can offer researchers a more comprehensive understanding of the research in their field, there is a lack of systematic analysis of literature related to ferroptosis and pyroptosis in PD. Therefore, this study applied bibliometric methods to systematically summarize the current research status and hotspots in the fields of ferroptosis in PD and pyroptosis in PD from the past to February 16, 2024. Furthermore, it discussed the possible future research trends, thereby providing the literature data support for the research on the mechanism of ferroptosis and pyroptosis in PD and the development of drugs targeting key molecules of ferroptosis and pyroptosis.

## 2 Materials and methods

### 2.1 Data source

**Figure 1:**
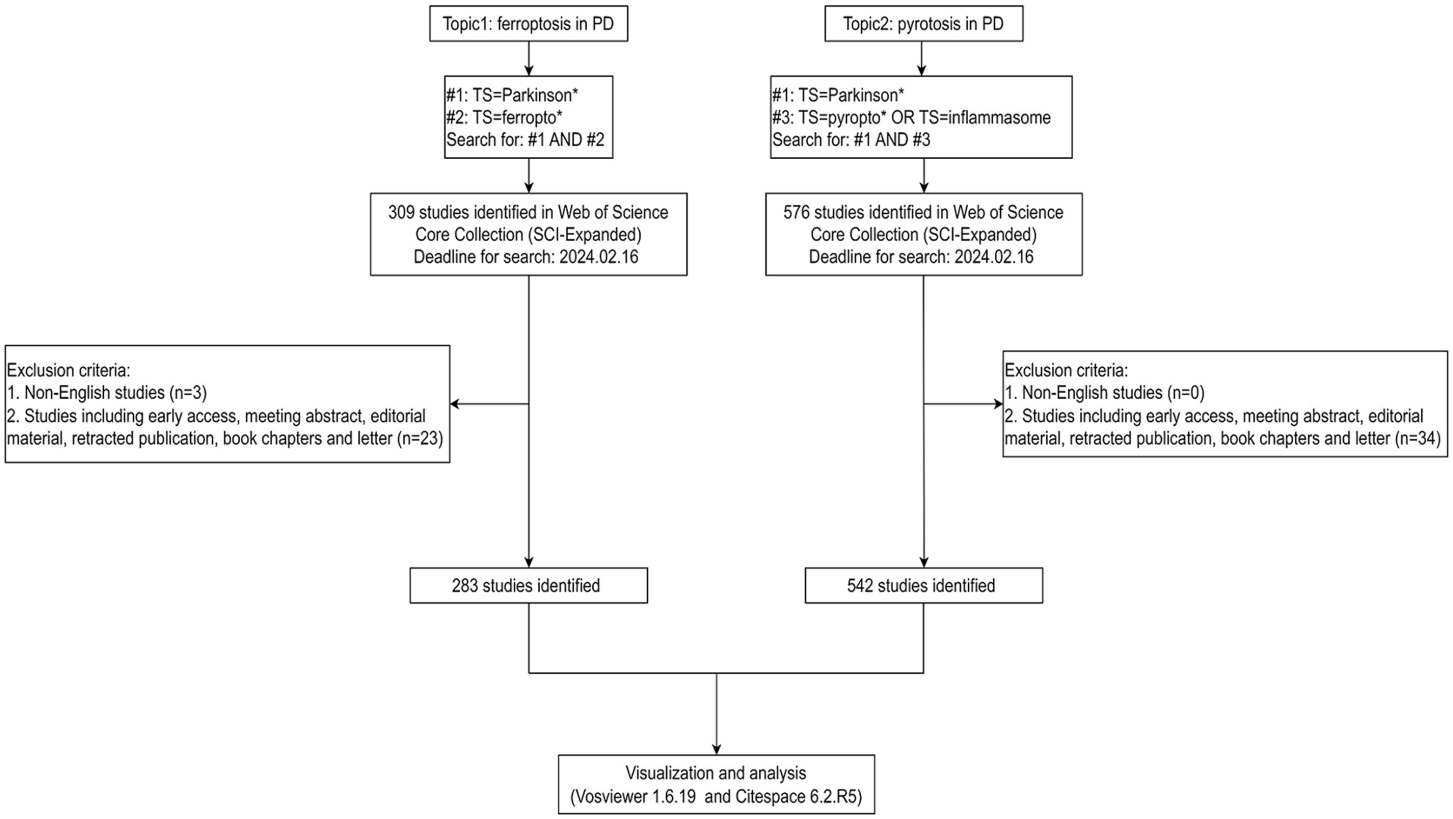
Document selection and flow diagram.

All data were obtained from the Science Citation Index Expanded (SCI-EXPANDED) in the Web of Science Core Collection (WoSCC) database. The following search strategy was used: #1 TS=Parkinson*, #2 TS = ferropto*, and #3 TS = pyropto* OR TS = inflammasome were defined, and then searches were conducted combining #1 AND #2, and #1 AND #3, respectively. The deadline for search was February 16, 2024. The methodology for data retrieval and collection is shown in Figure 1. The included documents were exported in plain text format with complete records and references, and saved as “download-txt.”

### 2.2 Statistical methods

Bibliometric and visualization analyses were performed using Vosviewer 1.6.19 and Citespace 6.2.R5. Focusing on the graphical depiction of bibliometrics, Vosviewer was utilized for visualizing the collaborations of countries/regions, institutions, authors and journals, co-citations of authors, journals, and references as well as the co-occurrence of keywords. Citespace can focus on extracting prospective data in the literature, so it was used for burst detection in references, dual-overlay analysis of journals, clustering analysis and time zone analysis of keywords.

## 3 Results

### 3.1 Annual publication volume and trend analysis

**Figure 2:**
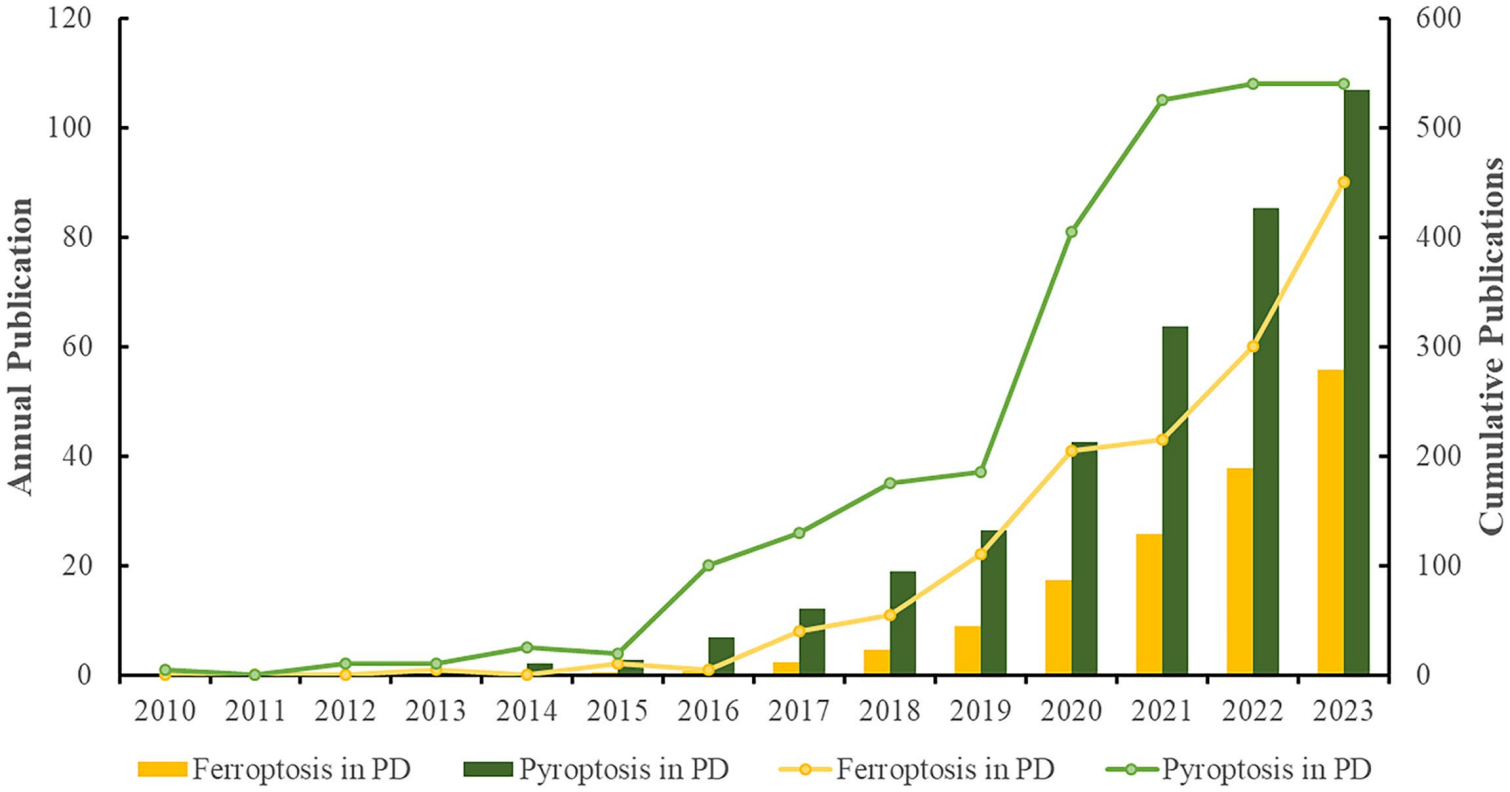
Analysis of annual research output (The line graphs depict the annual publication trends across two distinct domains, while the bar charts illustrate the cumulative volume of publications within these fields).

The number of publications in each period reflects the trend of research in the field. As shown in Figure 2, the research on “pyroptosis in PD” started earlier, with the first publication in 2010, which described the initiators and signaling pathways of inflammasome activation and its precise role in the pathogenesis of CNS inflammation (Chakraborty et al., 2010). The first study on “ferroptosis in PD” was published in 2013, which discussed in depth that the HIF prolyl hydroxylase could be an emerging target for iron chelators to inhibit ferroptosis in primary neurons and repair neuronal cell degeneration (Speer et al., 2013). Relevant studies in both fields have been reported gradually since then. Publication outputs in both areas were extremely low from the publication of the first relevant study until 2015, and the research remained stagnant, indicating that “ferroptosis in PD” and “pyroptosis in PD” were still in their infancy and had not attracted widespread attention. From 2016 to 2019, the volume of literature grew slowly in both areas, but the number of studies on “pyroptosis in PD” was higher than that on “ferroptosis in PD.” From 2020 to 2021, the research on “pyroptosis in PD” grew rapidly, while the research on “ferroptosis in PD” grew more slowly, indicating that “pyroptosis in PD” was the hotspot of the research in this stage, and the research focused on the development of a variety of PD therapeutics targeting molecules involved in the NLRP3 inflammatory pathway and the pyroptosis pathway, and much progress had been made in this phase. From 2022 to 2023, the research on “ferroptosis in PD” increased rapidly, while the research on “pyroptosis in PD” slowed down, indicating that more researchers were focusing on “ferroptosis in PD,” making it the current research hotspot, and the research mainly focused on the novel ferroptosis-related genes and molecular patterns in PD, which provided new directions for finding new therapeutic targets and early diagnostic specific biomarkers. The data collection up to February 2024 were insufficient, and including the 2024 data in the figure could negatively impact the trend analysis. Therefore, it will not be displayed in the graph.

### 3.2 Country/region analysis

A total of 43 and 50 countries were involved in the study of “ferroptosis in PD” and “pyroptosis in PD,” respectively. Table 1 summarizes the top 10 productive countries/regions in these two fields. China and the USA were the top two countries in both the number of publications and citations in these two fields, ahead of other countries, indicating their significant contributions. It was interesting to note that Japan, ranked 9th in publication volume, had the highest average citation in the field of “ferroptosis in PD,” with Italy having the highest average number of citations in the field of “pyroptosis in PD,” which may be linked to the higher quality of their articles.

**Table 1:** Top 10 countries/regions related to ferroptosis, pyroptosis in Parkinson’s disease.

Type	Rank	Country/region	Documents	Citations	Total link strength	Average citation
Ferroptosis in PD	1	China	158	8,873	25	56.16
	2	USA	45	5,651	30	125.58
	3	Australia	19	5,673	20	298.58
	4	England	16	972	23	60.75
	5	Germany	16	4,936	12	308.50
	6	France	13	1,103	17	84.85
	7	Italy	11	66	5	6.00
	8	India	10	103	7	10.30
	9	Japan	9	4,199	5	466.56
	10	Netherlands	7	245	8	35.00
Pyroptosis in PD	1	China	274	7,982	34	29.13
	2	USA	114	5,219	55	45.78
	3	Italy	39	2,599	14	66.64
	4	England	20	1,275	15	63.75
	5	India	19	395	6	20.79
	6	Australia	18	1,070	12	59.44
	7	South korea	17	898	8	52.82
	8	Canada	15	273	14	18.20
	9	Germany	15	523	14	34.87
	10	Spain	15	358	10	23.87

**Figure 3:**
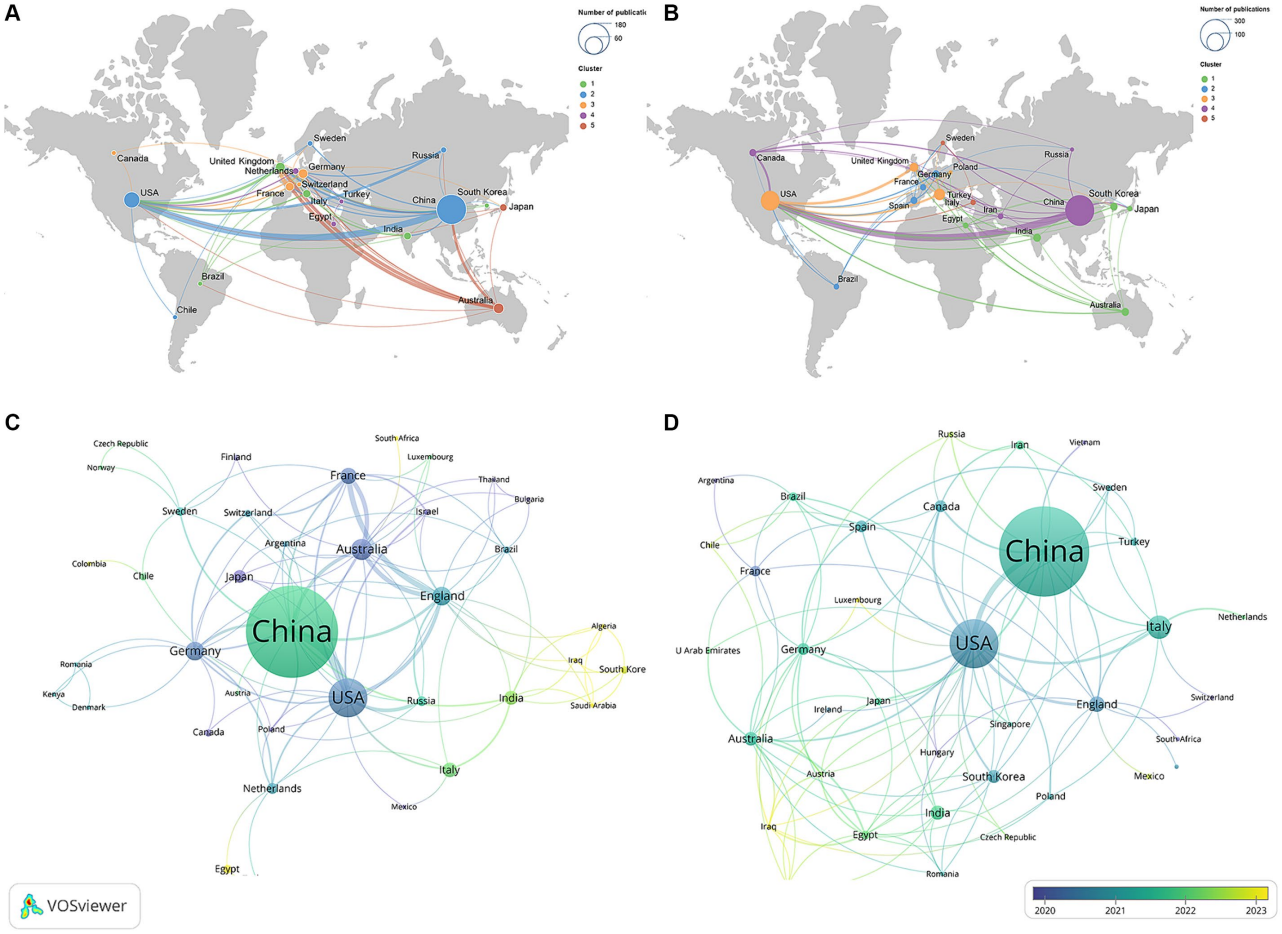
Geographical collaboration map related to **(A)** “ferroptosis in PD”, **(B)** “pyroptosis in PD” and time visualization of countries related to **(C)** “ferroptosis in PD,” **(D)** “pyroptosis in PD”.

The geographic collaboration network between countries is presented in Figures 3A,B. The size of the nodes indicated the publication outputs, the color of the nodes indicated the cooperation clusters, while the line thickness between two nodes indicated the strength of the cooperation, i.e., the stronger the Total Link Strength (TLS). The top three countries in terms of TLS in these two domains were the USA, China, and England, indicating their active cooperation and exchanges in the field, which promoted the development of this field.

Figures 3C,D show the temporal view of the countries, and the gradient color from purple to yellow represented the freshness of publications. Countries such as Japan, Canada, Poland in the field of “ferroptosis in PD” and Luxembourg, Iraq in the field of “pyroptosis in PD” were marked in a dark purple, indicating that these countries had participated in the research in this field earlier, providing a historical backdrop for current trends. In contrast, countries displayed in a dark yellow, such as Iraq, Algeria, and Saudi Arabia in the field of “ferroptosis in PD” and Switzerland, Vietnam, and Argentina in the field of “pyroptosis in PD,” indicating that these countries recently contributed some new publications, which had contributed to the progress of this research field at this stage.

### 3.3 Institutional analysis

A total of 474 and 727 institutions participated in research on “ferroptosis in PD” and “pyroptosis in PD” respectively, with the majority being comprehensive universities and medical universities. The 10 institutions with the highest productivity in the two fields are listed in Table 2. Among the top 10 institutions, 6 belonged to China, and the University of Melbourne in Australia ranked first in terms of the number of publications, citation frequency, and TLS in the field of “ferroptosis in PD.” Similarly, in the field of “pyroptosis in PD,” 8 of the top 10 institutions for publications were from China, and Nanjing Medical University of China ranked first in terms of publications, citations, and TLS, hinting at their broad influence in this field.

**Table 2:** Top 10 institutions related to ferroptosis, pyroptosis in Parkinson’s disease.

Type	Rank	Institutions	Documents	Citations	Average citation	Total link strength
Ferroptosis in PD	1	Univ Melbourne	15	5,604	373.60	58
	2	Qingdao Univ	9	232	25.78	1
	3	Chinese Acad Sci	8	247	30.88	20
	4	Lille Univ	8	630	78.75	28
	5	Sichuan Univ	8	685	85.63	18
	6	China Med Univ	7	72	10.29	3
	7	Guangzhou Univ Chinese Med	7	241	34.43	9
	8	Univ Groningen	7	245	35.00	16
	9	Univ Cambridge	6	427	71.17	30
	10	Zhejiang Univ	6	96	16.00	4
Pyroptosis in PD	1	Nanjing Med Univ	28	1,444	51.57	38
	2	Nanjing Univ Chinese Med	18	1,050	58.33	29
	3	Huazhong Univ Sci & Technol	11	375	34.09	17
	4	Qingdao Univ	10	303	30.30	17
	5	Univ Queensland	10	605	60.50	14
	6	Zhejiang Univ	10	178	17.80	5
	7	China Med Univ	9	229	25.44	11
	8	Iowa State Univ	9	991	110.11	14
	9	Shandong Univ	9	178	19.78	8
	10	Chinese Acad Sci	8	282	35.25	33

**Figure 4:**
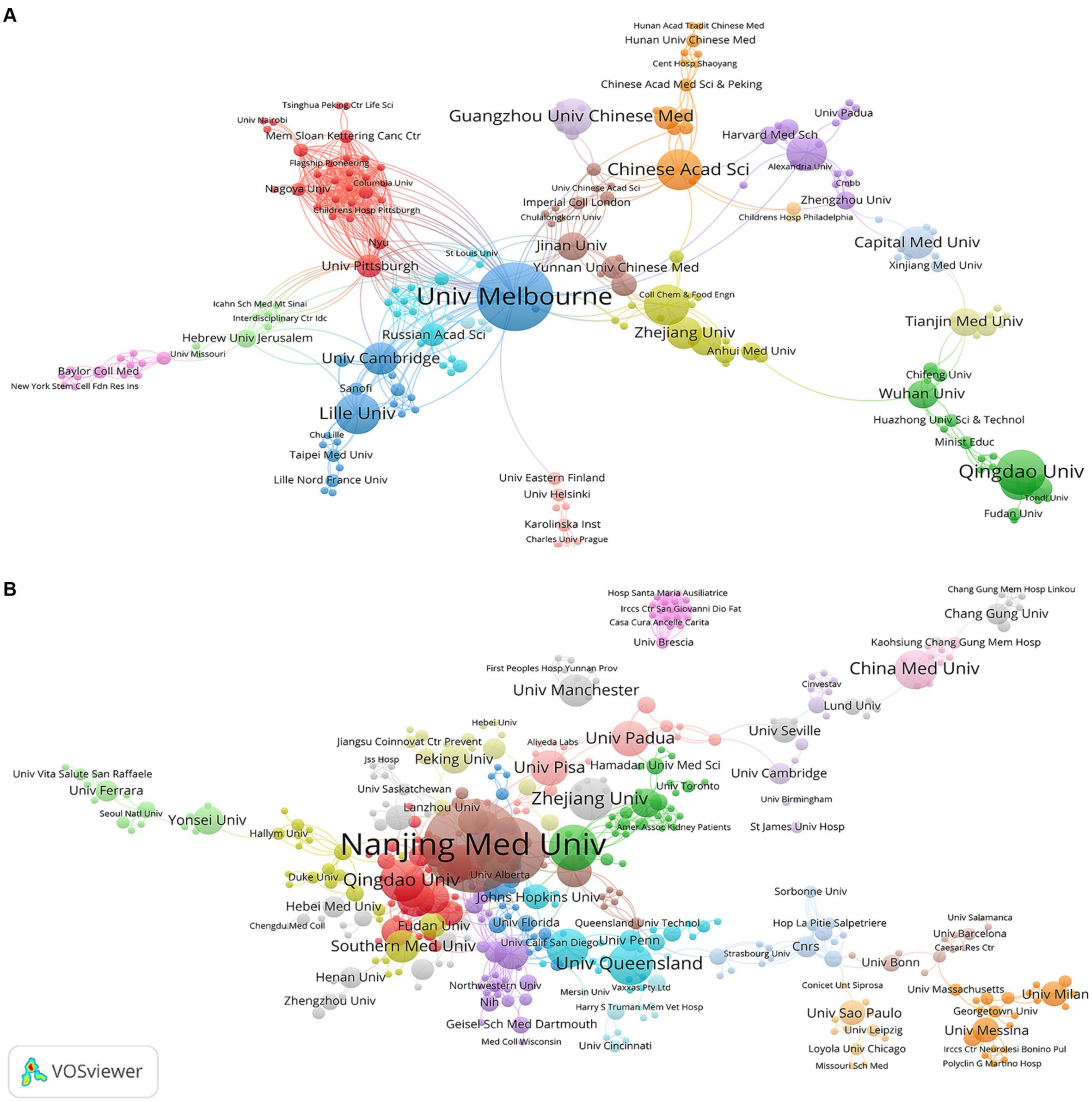
Visualization map of institutions related to **(A)** “ferroptosis in PD”, **(B)** “pyroptosis in PD”.

Figure 4 shows the mapping of the collaboration network among institutions, respectively. Univ Melbourne (TLS = 58), and Nanjing Med Univ (TLS = 38) were the institutions with the strongest TLS for “ferroptosis in PD” and “pyroptosis in PD,” respectively. They were key hubs for promoting international collaboration among related research institutions, with the highest value of centrality. However, there were obvious regional limitations to institutional cooperation. Therefore, for greater breakthroughs in two fields, it is highly advised that regional and disciplinary barriers should be broken down and cooperation and exchanges should be conducted between all research institutions.

### 3.4 Authors and co-cited authors analysis

A total of 1,640 and 3,024 authors wrote articles on “ferroptosis in PD” and “pyroptosis in PD,” respectively. The top 10 most published and co-cited authors in the two fields are listed in Table 3. David Devos ranked first in the field of “ferroptosis in PD with 8 publications, followed by Meiling Zhu (*n* = 7) and Dongfeng Chen (*n* = 6). In the field of “pyroptosis in PD,” Gang Hu (*n* = 16), Ming Lu (*n* = 16), Anumantha G Kanthasamy (*n* = 9) ranked in the top 3, with more than 50% of the top 10 authors in both fields being from China, demonstrating a similar trend with the distribution of leading institutions. The above statistics underscored China’s prominent role in disseminating related research in the fields.

**Table 3:** Top 10 authors and co-cited authors related to ferroptosis, pyroptosis in Parkinson’s disease.

Type	Rank	Author	Documents	Citations	Avg. pub. Year	Rank	Co-cited author	Citations
Ferroptosis in PD	1	Devos, David	8	996	2020	1	Dixon, Sj	317
	2	Zhu, Meiling	7	241	2021	2	Yang, Ws	255
	3	Chen, Dongfeng	6	239	2021	3	Ayton, S	159
	4	Devedjian, Jean-Christophe	6	717	2020	4	Doll, S	155
	5	Ayton, Scott	5	559	2020	5	Stockwell, Br	150
	6	Li, Xinrong	5	233	2020	6	Do Van, B	135
	7	Liu, Xuelei	5	233	2020	7	Gao, Mh	132
	8	Xie, Junxia	5	48	2023	8	Angeli, Jpf	118
	9	Hao, Xiaoqian	4	200	2021	9	Devos, D	99
	10	Lei, Peng	4	589	2021	10	Kagan, Ve	82
Pyroptosis in PD	1	Hu, Gang	16	1,106	2019	1	Heneka, Mt	308
	2	Lu, Ming	16	1,122	2019	2	Gordon, R	166
	3	Kanthasamy, Anumantha G.	9	991	2019	3	Codolo, G	133
	4	Qiao, Chen	9	728	2017	4	Lee, E	124
	5	Chen, Ying	8	144	2020	5	Yan, Yq	124
	6	Anantharam, Vellareddy	7	502	2019	6	Martinon, F	123
	7	Ding, Jian-Hua	7	694	2016	7	Sarkar, S	108
	8	Jin, Huajun	7	502	2019	8	Zhou, Y	108
	9	Kanthasamy, Arthi	7	502	2019	9	Zhou, Rb	107
	10	Sarkar, Souvarish	7	525	2019	10	He,Y	105

**Figure 5:**
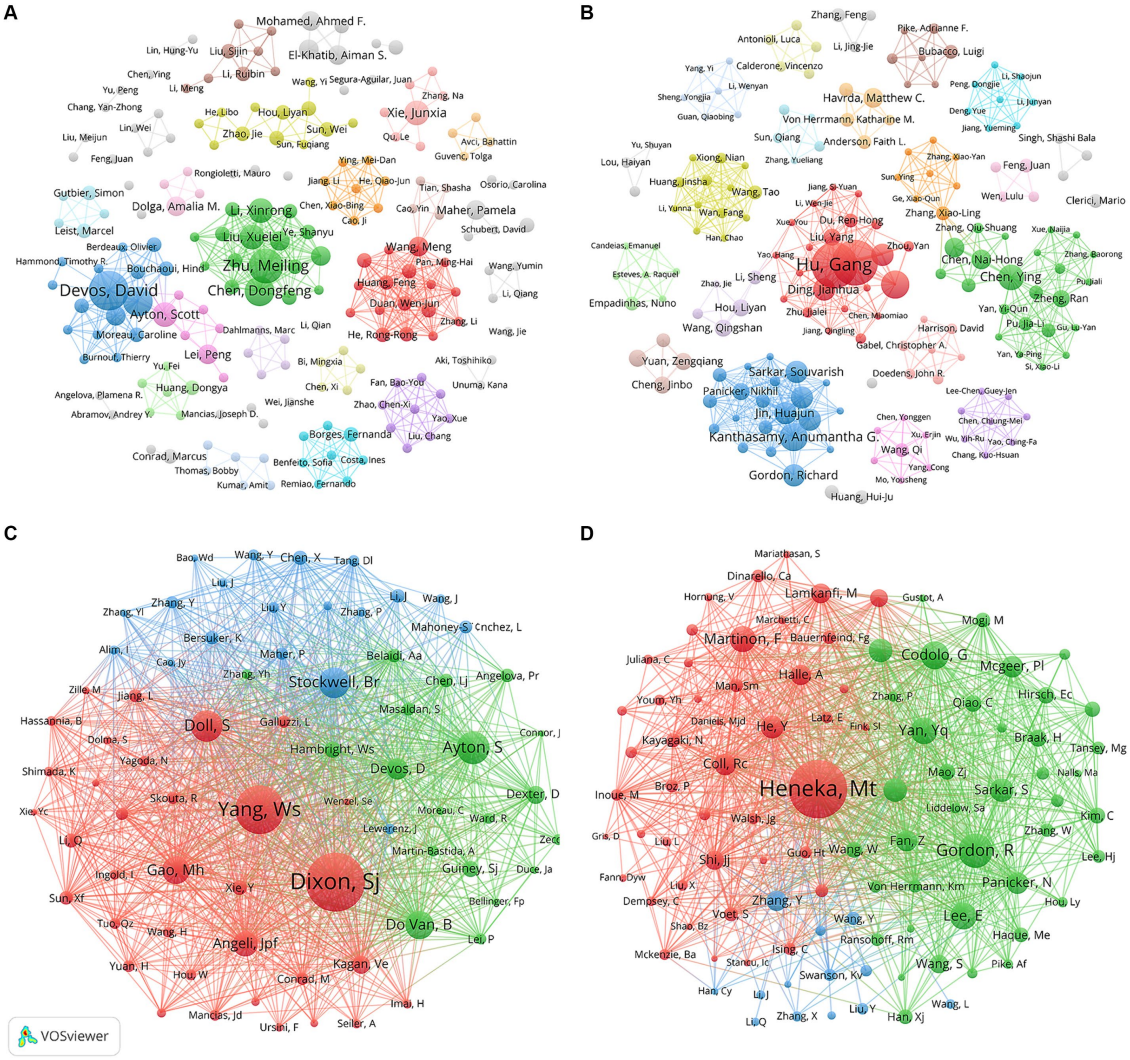
Visualization map of co-authorship related to **(A)** “ferroptosis in PD”, **(B)** “pyroptosis in PD” and co-cited authorship related to **(C)** “ferroptosis in PD”, **(D)** “pyroptosis in PD”.

Figures 5A,B describe the collaborative networks of the authors of “ferroptosis in PD” and “pyroptosis in PD,” respectively. The different colors represented collaboration clusters of authors, and the lines between nodes indicated the strength of their cooperation. Among these clusters, main partnerships and outstanding researchers could be discovered. In the field of “ferroptosis in PD,” the three most significant cooperation clusters were identified, namely red, green, and blue clusters. The green clusters included Meiling Zhu, Dongfeng Chen, Xuelei Liu and Xinrong Li. In the field of “pyroptosis in PD,” three essential collaborative clusters had been formed, led by Gang Hu, Anumantha G Kanthasamy and Ying Chen, respectively, who were also among the top in publication volume, indicating that the main research authors had formed a collaborative network. However, most of the collaborations between authors were within national boundaries, suggesting that international exchange and collaboration among authors from various countries should be strengthened to produce higher-quality academic outcomes in the field.

A total of 16,486 and 27,946 co-cited authors contributed to the publication of “ferroptosis in PD” and “pyroptosis in PD,” respectively. Scott J Dixon had been co-cited 317 times, ranked the first in the field of “ferroptosis in PD,” followed by Wan Seok Yang (255 citations) and Scott Ayton (159 citations) (Figure 5C). Simultaneously, two authors, Scott Ayton and David Devos were among the top 10 in both publication volume and co-citation frequency, highlighting their significant role and influence as core leaders in this domain. In the area of “pyroptosis in PD,” the top three authors in terms of co-citations were Michael Heneka (308 citations), followed by Richard Gordon (166 citations) and Gaia Codolo (133 citations) (Figure 5D).

### 3.5 Journals and co-cited journals analysis

**Figure 6:**
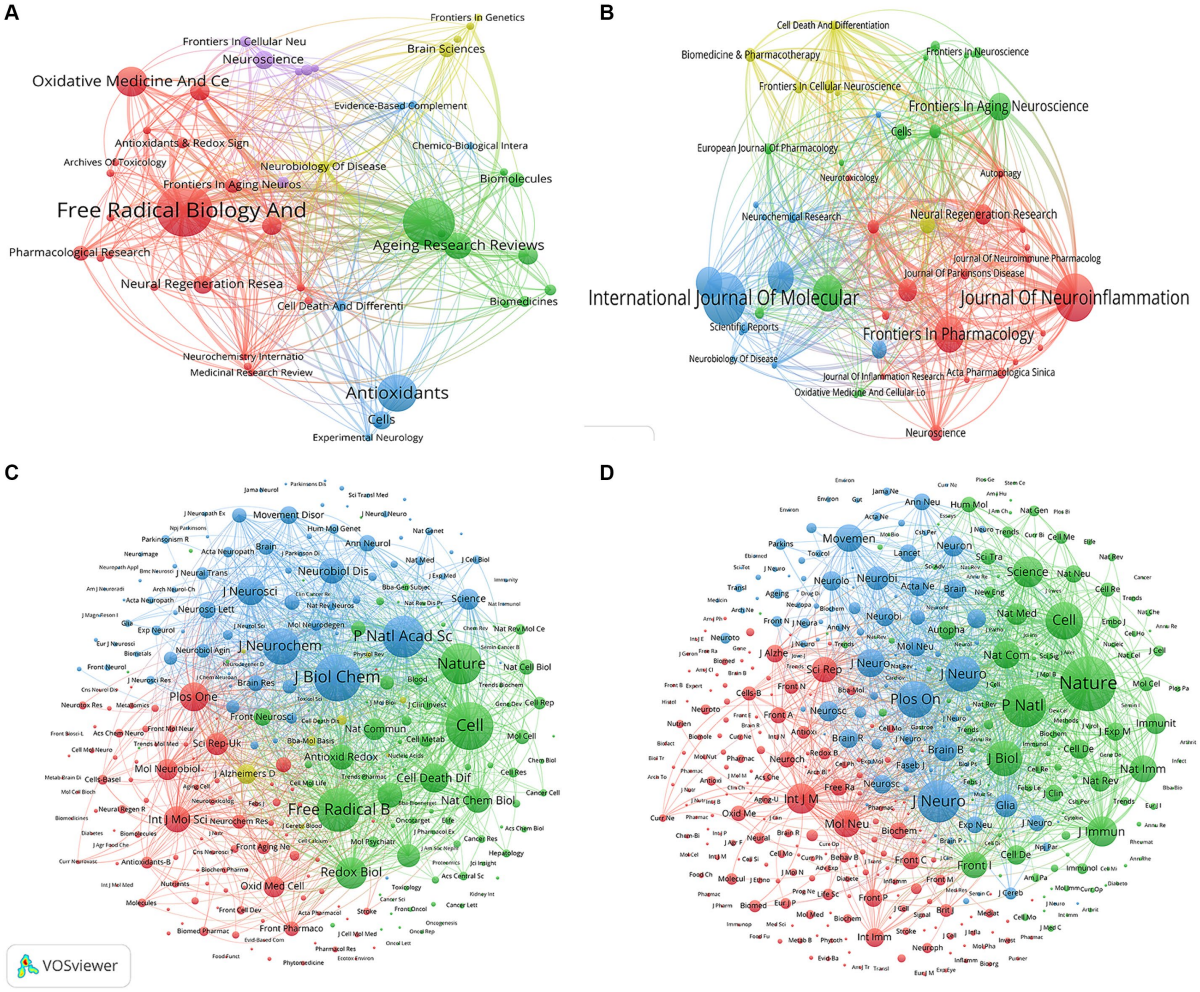
Visualization map of journals related to **(A)** “ferroptosis in PD”, **(B)** “pyroptosis in PD” and co-cited journals related to **(C)** “ferroptosis in PD”, **(D)** “pyroptosis in PD”.

A total of 158 and 241 academic journals published articles in the fields of “ferroptosis in PD” and “pyroptosis in PD.” The top 10 most published and co-cited journals in the two fields are listed in Supplementary Table S1. *Free radical biology and medicine* (*n* = 14, IF = 7.4) and *International journal of molecular sciences* (*n* = 23, IF = 5.6) ranked first in publications on “ferroptosis in PD” and “pyroptosis in PD” (Figures 6A,B). Meanwhile, co-citation analysis revealed that the journals with the highest co-citations were *Cell* (*n* = 829) and *Nature* (*n* = 1,651) in the fields of “ferroptosis in PD” and “pyroptosis in PD,” respectively. (Figures 6C,D), indicating the high academic influence of articles published by the journals. Among the top 10 journals in terms of number of publications and co-citation frequency, most of the journals were Q1 journals, with *Nature* having the highest IF of 64.8. The relevance between high IFs and citation frequency emphasizes the significance of these journals in advancing the development of this topic.

**Figure 7:**
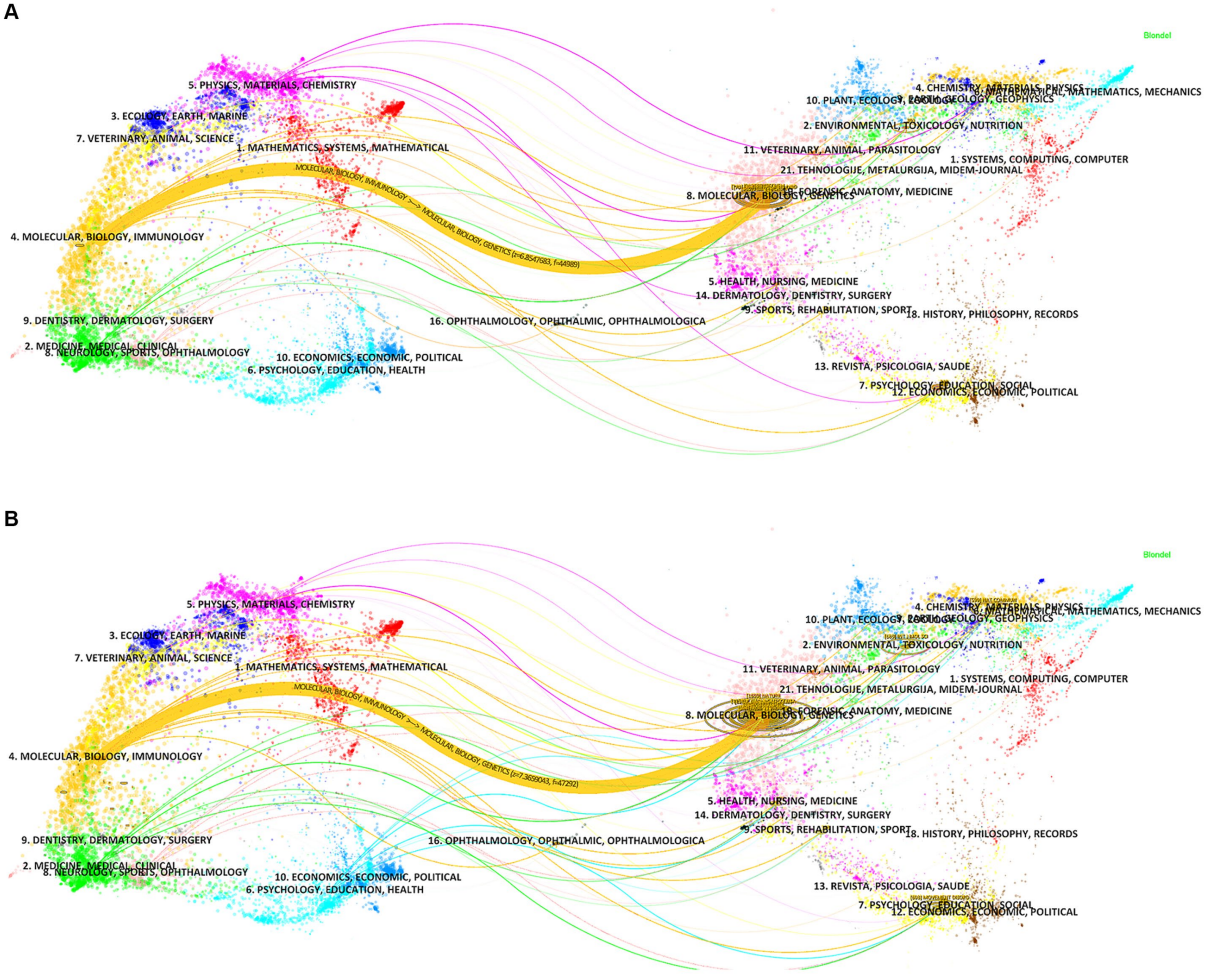
The dual-map overlay of journals related to **(A)** “ferroptosis in PD”, **(B)** “pyroptosis in PD”.

The dual-map overlay analysis of journals (Figure 7) was made by Citespace, showing the distribution of different journal themes, depicting the various research areas covered by all journals, with colored paths representing citation relationships, and clusterings of the citing journals on the left and cited journals on the right. A primary orange citation path was identified, demonstrating that articles in the Molecular, Biology and Genetics journals for the studies “ferroptosis in PD” and “pyroptosis in PD” were mainly cited by articles in the Molecular, Biology and Immunology journals. This implies that research in these two areas requires interdisciplinary collaboration.

### 3.6 Co-cited references analysis

**Figure 8:**
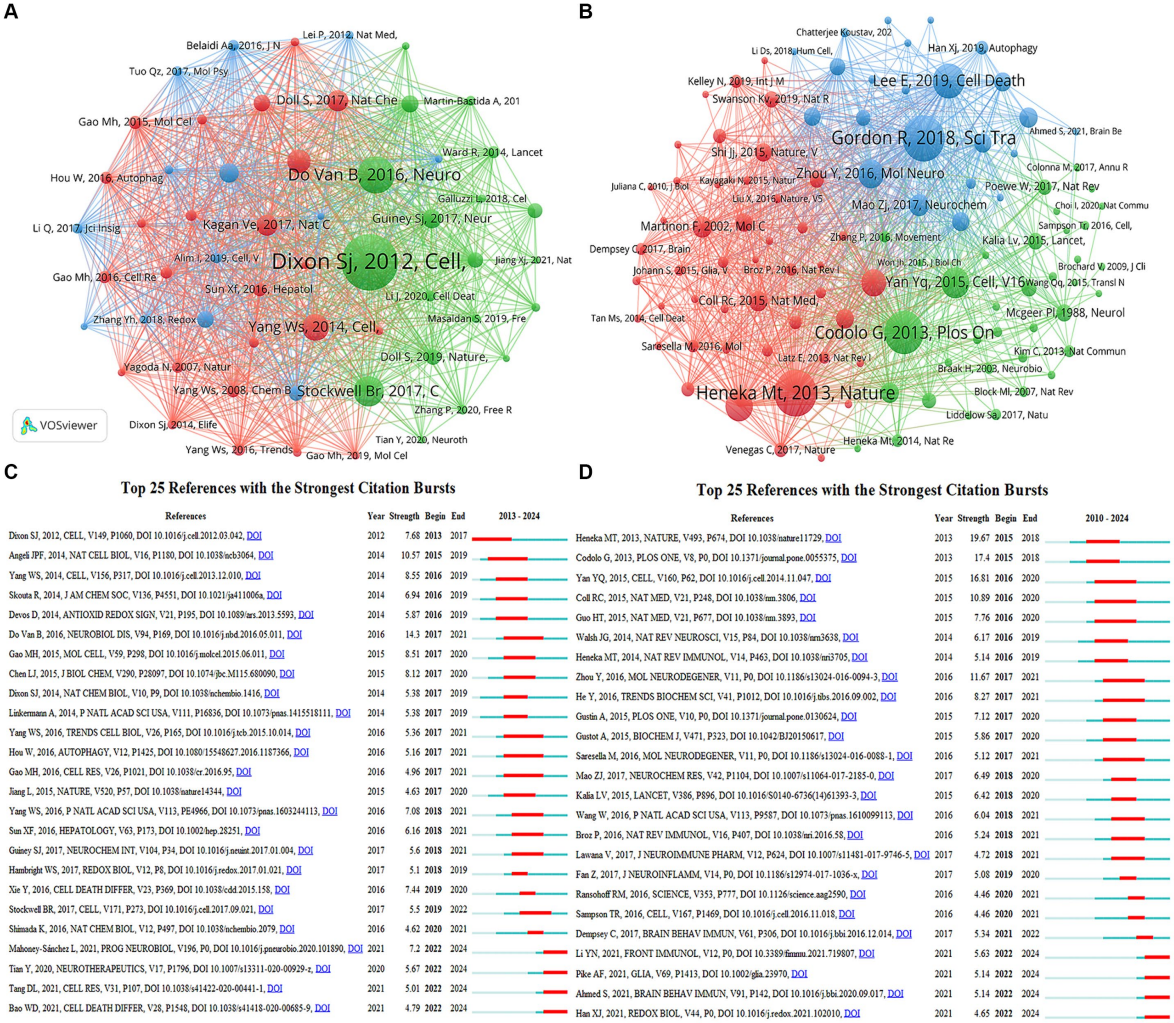
Visualization of co-citation references related to **(A)** “ferroptosis in PD”, **(B)** “pyroptosis in PD”. The top 25 references with the strongest citation bursts in researches related to **(C)** “ferroptosis in PD”, **(D)** “pyroptosis in PD”.

Co-cited references are two or more references that are simultaneously cited by another paper, forming a co-citation relationship. Supplementary Table S2 displays the details of the top 10 most frequently co-cited references in the two fields. As shown in Figure 8A, Scott J Dixon’s article “Ferroptosis: an iron-dependent form of nonapoptotic cell death” (Dixon et al., 2012) published in *Cell* was the most co-cited reference in the field of “ferroptosis in PD,” which named and established ferroptosis as a novel modality of regulated cell death, a seminal discovery that had a major impact on subsequent research in this field. Following this is Bruce Do Van’s article “Ferroptosis, a newly characterized form of cell death in Parkinson’s disease that is regulated by PKC” (Do Van et al., 2016) published in *Neurobiology of Disease*, highlighting the role of ferroptosis in PD, revealing its activation through a PKCα-mediated, RAS-independent mechanism and iron chelators, Fer-1, and PKC inhibitors prevent neuronal loss in PD patients, providing new insights into PD treatment. As shown in Figure 8B, Richard Gordon’s article “Inflammasome inhibition prevents α-synuclein pathology and dopaminergic neurodegeneration in mice” (Gordon et al., 2018) was the most frequently cited article in the field of “pyroptosis in PD,” revealing that NLRP3 in microglia may persistently cause neuroinflammation to drive progressive DA neurons loss, and emphasizing NLRP3 as a potential therapeutic target in PD, opening a new window for PD research. Predominantly, these key articles were published in reputable journals such as *Cell* and *Nature*, with *Cell* particularly including three articles of the top 10 in the area of “ferroptosis in PD.” These publications covered a range of themes related to ferroptosis in PD, including regulation, molecular mechanisms, targeted inhibition and PD therapy.

Emerging references are those whose citation frequency suddenly increases significantly within a short period, indicating research hotspots and frontier trends at that time. Figures 8C,D show the top 25 references with the strongest citation bursts in the two fields, respectively. In “ferroptosis in PD,” Bruce Do Van published “Ferroptosis, a newly characterized form of cell death in Parkinson’s disease that is regulated by PKC” (Do Van et al., 2016) had the highest burst strength of 14.3, with an burst duration of 2017–2021. Four references continued to exhibit citation bursts until 2024, describing the morphological features, regulatory mechanisms, and signaling pathways of ferroptosis, its inducers and inhibitors, and its role in diseases. In “pyroptosis in PD,” “NLRP3 is activated in Alzheimer’s disease and contributes to pathology in APP/PS1 mice” (Heneka et al., 2013) had the highest burst strength of 19.67, with a burst period of 2015–2018. Four references continued to exhibit citation bursts until 2024, describing the specific roles and interactions between GSDM family members and the family of inflammatory caspases in the process of pyroptosis, further elucidating the relationship between pyroptosis and PD, and providing new strategies for research into pyroptosis-mediated PD.

### 3.7 Keyword analysis

**Figure 9:**
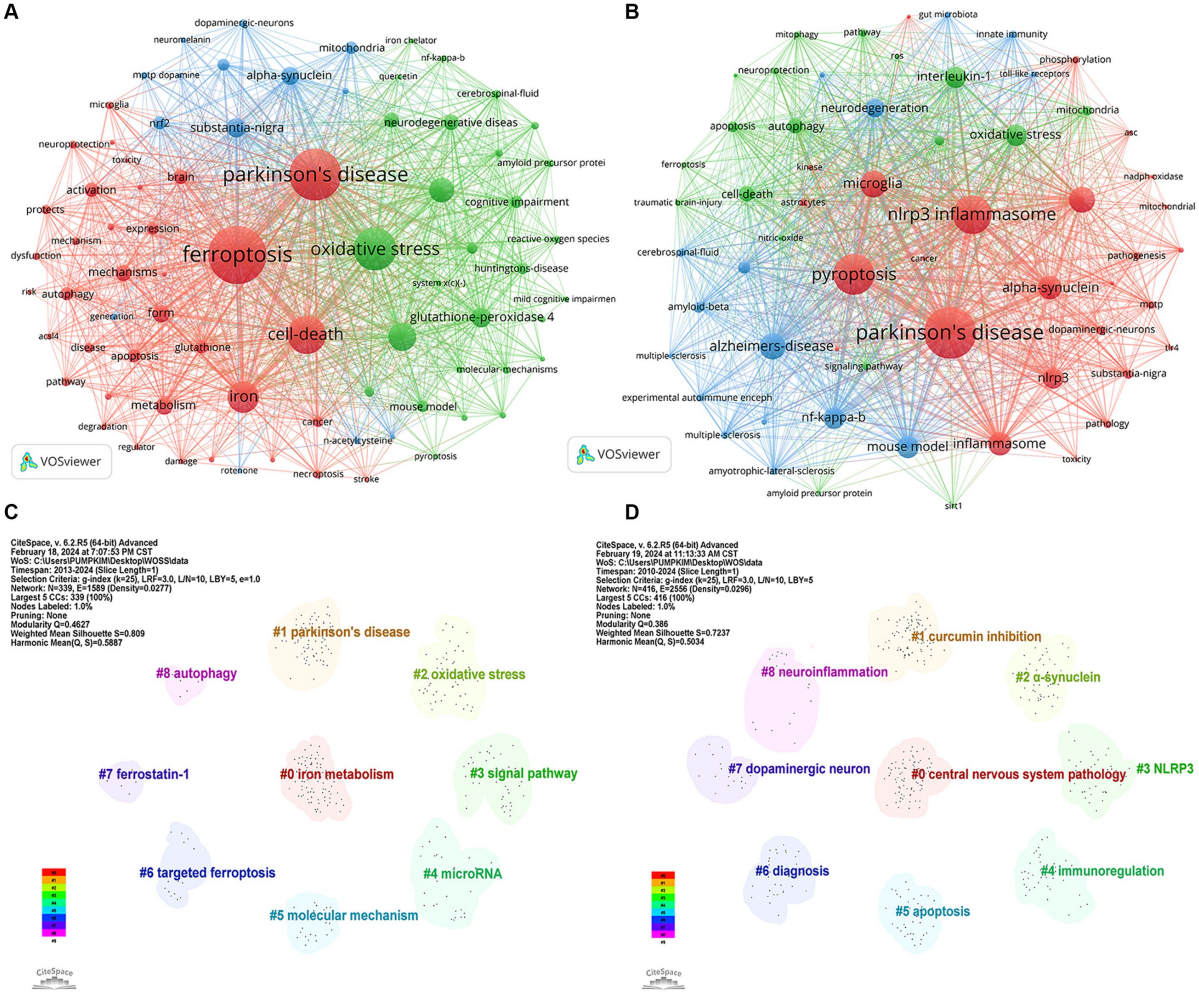
Visualization of keywords related to **(A)** “ferroptosis in PD”, **(B)** “pyroptosis in PD” and keyword clusters related to **(C)** “ferroptosis in PD”, **(D)** “pyroptosis in PD”.

Keywords represent a high-level summary and refinement of article content, through keyword co-occurrence, it is possible to understand the hotspot and development trend of the current research field. Table 4 shows the basic information of the top 15 most frequent keywords in the two fields. It can be seen from Figure 9A that the top 5 keywords with a high occurrence frequency of “ferroptosis in PD” were “ferroptosis” “Parkinson’s disease” “oxidative stress” “cell death” and “iron.” From the results, indicating research focused on diseases like “Parkinson’s disease” “stroke” “Alzheimer’s disease” and “cancer”; pathological mechanisms including “oxidative stress” “lipid peroxidation” “glutamate” “ferroptosis” “autophagy” “apoptosis” “pyroptosis” and “reactive oxygen species”; drug research represented by “vitamin e” “acetylcysteine” and “quercetin”; and signaling pathways focusing on “Nrf2/GPX4” and “cystathionine-glutamate transporter” (Xct). As shown in Figure 9B, the keywords appearing at a high frequency of “pyroptosis in PD” were “Parkinson’s disease” “pyroptosis” “nlrp3 inflammasome” “microglia” and “Alzheimer’s-disease.” From the results, we can see that research focused on diseases like “Parkinson’s disease” “Alzheimer’s disease” and “traumatic brain injury”; pathological mechanisms including “cell death” “pyroptosis” “ferroptosis” “autophagy” “apoptosis” “pyroptosis” “microglia” “nlrp3 inflammasome” and “α-synuclein”; and signaling pathways focusing on “NF-kappa-B” “NLRP3” “interleukin-1” and “caspase-1.”

**Table 4:** Top 15 occurrences keywords associated with ferroptosis, pyroptosis in Parkinson’s disease.

Type	Rank	Keyword	Occurrences	Total link strength
Ferroptosis in PD	1	ferroptosis	200	1,159
	2	parkinson’s disease	170	1,005
	3	oxidative stress	126	785
	4	cell-death	108	675
	5	iron	81	525
	6	lipid-peroxidation	67	461
	7	alzheimers-disease	56	383
	8	glutathione-peroxidase 4	40	299
	9	substantia-nigra	39	267
	10	alpha-synuclein	35	240
	11	form	32	173
	12	metabolism	32	176
	13	mechanisms	30	176
	14	neurodegenerative diseases	27	200
	15	mitochondrial dysfunction	26	159
Pyroptosis in PD	1	parkinson’s disease	351	1,586
	2	pyroptosis	245	1,212
	3	nlrp3 inflammasome	225	1,038
	4	microglia	128	660
	5	alzheimers-disease	125	675
	6	capase-1	125	639
	7	alpha-synuclein	107	570
	8	inflammasome	104	511
	9	mouse model	95	509
	10	interleukin-1	93	448
	11	oxidative stress	93	420
	12	nf-kappa-b	91	464
	13	nlrp3	87	451
	14	neurodegeneration	75	415
	15	autophagy	55	274

Based on the keyword co-occurrence analysis, the keywords with similar features were clustered, reflecting the basic knowledge structure of the related research fields. In CiteSpace, the modularity (Q) value and silhouette (S) value, are calculated based on the network structure and the clarity of the clustering. Generally, Q > 0.3 indicates a significant clustering structure; when S > 0.5, the clustering is deemed reasonable, and when S > 0.7, the clustering is considered credible. Figure 9C identified nine relevant clusters of “ferroptosis in PD”, ranging from #0 (iron metabolism) to #8 (autophagy), as delineated by CiteSpace, with a Q value of 0.4627 and an S value of 0.809, reinforcing remarkable network homogeneity and clustering structure. Among them, #0 iron metabolism, #5 molecular mechanism, #3 signal pathway, #2 oxidative stress, and #4 microRNA mainly explored the regulatory mechanism of ferroptosis in PD. #1 Parkinson’s disease was the exploration of related diseases. #7 ferrostatin-1 and #6 targeted ferroptosis explored therapeutic strategies. #8 autophagy explored other forms of cell death

As shown in Figure 9D, the keywords of “pyroptosis in PD” were clustered into 9 clusters, ranging from #0 (CNS diseases) to #8 (neuroinflammation), with a Q value of 0.386 and an S value of 0.7237, indicating distinct research themes and convincing clustering. Among them, #2 α-synuclein, #3 NLRP3, #7 dopaminergic neuron, and #8 neuroinflammation mainly explored the regulatory mechanism of pyroptosis in PD. #0 central nervous system pathology focused on related disorders. #1 curcumin inhibition, #6 diagnosis explored the diagnostic modality and treatment in traditional Chinese medicine. #5 apoptosis explored other forms of cell death.

**Figure 10:**
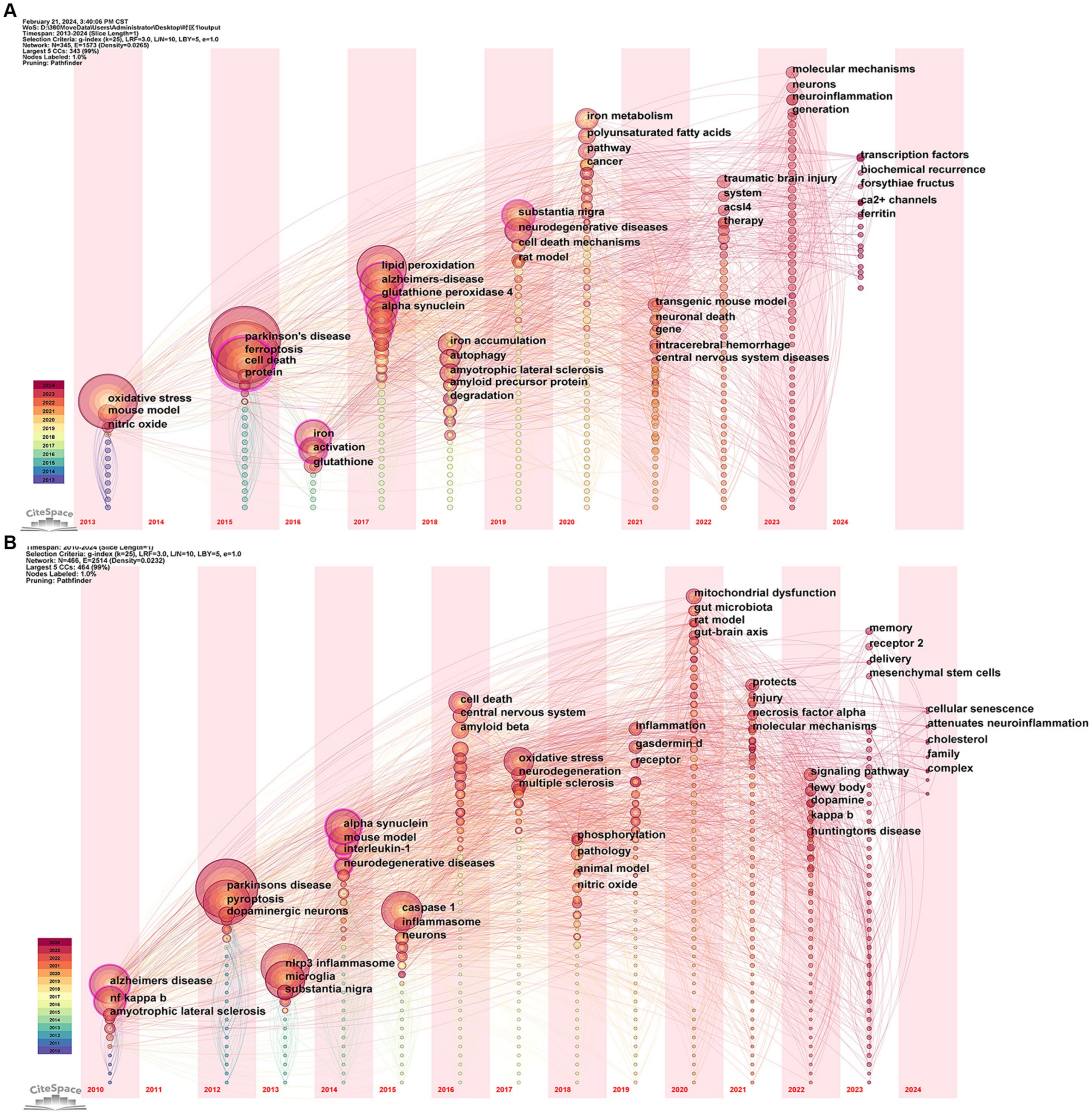
Visualization of the keyword time zone related to **(A)** “ferroptosis in PD”, **(B)** “pyroptosis in PD”.

The keyword time zone map is used to identify research themes from various periods to analyze the evolutionary lineage and predict the direction of development. Figure 10A shows that in the field of “ferroptosis in PD,” the earliest appearing keywords were “oxidative stress,” “mouse model,” and “nitric oxide,” which were the basis of the research in this field and provide historical background for the current research trends. In 2023, research mainly focused on “molecular mechanisms,” “neurons,” “neuroinflammation” and “generation,” which may be the current hotspots in the field. The research already touched upon in 2024 included “ferritin,” “Ca^2+^ channels,” and “forsythia fructus.” As shown in Figure 10B, in the area of “pyroptosis in PD,” the earliest appearing keywords included “Alzheimer’s disease,” “NF kappa B,” and “amyotrophic lateral sclerosis.” The research in 2023 focused on “receptor 2,” “mesenchymal stem cell,” and “delivery,” marking them as hotspots in the field. The research mentioned for 2024 included “cholesterol,” “attenuates neuroinflammation,” and “cellular senescence.” The research involved in 2024 cannot conclusively prove to be a trend or hotspot, with insufficient data resulting from the early cutoff of literature retrieval.

## 4 Discussion

### 4.1 General information

The study used bibliometric tools to analyze the literature in the fields of “ferroptosis in PD” and “pyroptosis in PD.” These fields are found to be on the rise, especially “ferroptosis in PD,” which has become a hot research topic in recent years and may become a future research trend.

In the analysis of countries/regions and institutions, China and the United States ranked the top two in the number of publications in the fields of “ferroptosis in PD” and “pyroptosis in PD,” which were far ahead of other countries/regions, highlighting the leading position of these two countries in the above research fields. Moreover, more than 60% of the top 10 institutions in both fields were from China, demonstrating that the above fields had attracted widespread attention in China, and the University of Melbourne and Nanjing Medical University were the leaders in terms of the number of documents, citation frequency and centrality of “ferroptosis in PD” and “pyroptosis in PD,” respectively. However, a dispersed layout among some countries and institutions were evident, with international collaborations being notably less common compared to domestic ones. Thus, constructing a robust network of cooperation represents a key challenge for further advancements in the two fields.

In the analysis of authors and co-cited authors, in the field of “ferroptosis in PD,” David Devos was the most prolific author, with a notable collaboration among high-publishing authors from Guangdong University of Traditional Chinese Medicine such as Meiling Zhu, Dongfeng Chen, Xuelei Liu, and Xinrong Li. For example, they discovered in 2021 that miR-335 decreases FTH1 level leading to ferroptosis in PD model, providing new ideas and potential for the study of pharmacological targets of PD (Li X. R. et al., 2021). Scott J Dixon had the most co-cited frequency, whose 2012 article in *Cell* “Ferroptosis: an iron-dependent form of nonapoptotic cell death” distinguished ferroptosis from traditional apoptosis and necrosis and autophagy, identifying ferrostatin-1 as a potent ferroptosis inhibitor (Dixon et al., 2012). In the field of “pyroptosis in PD,” Gang Hu was the most published author, with close collaborations with other high-publication authors such as Ming Lu, Chen Qiao, and Jianhua Ding, demonstrating that Nlrp3 is a target gene of microRNA-7 (miR-7) (Zhou et al., 2016). Michael Heneka, with the highest co-citation frequency, had made important contributions to the mechanism of pyroptosis and the clinical application of PD therapy. The co-citation frequency of these people reflected their significant influence and recognition in the scientific community.

In the analysis of journals and co-cited journals, *Free radical biology and medicine* had the highest number of publications in the field of “ferroptosis in PD,” while *Cell* had the highest co-citation frequency. In the field of “pyroptosis in PD,” *International journal of molecular sciences* led in publication count with *Nature* having the highest number of co-citations. Most of these journals were in the Q1 JCR segment, especially the top journals such as *Nature* (IF = 64.8) and *Cell* (IF = 64.5), indicating the participation of numerous high-quality journals in this field, thus suggesting a promising research prospect. Overall, the findings will help researchers to quickly and accurately find appropriate journals for the latest advances in the above-mentioned fields.

In the analysis of co-cited references, “Ferroptosis: an iron-dependent form of nonapoptotic cell death” and “Inflammasome inhibition prevents α-synuclein pathology and dopaminergic neurodegeneration in mice” were the most cited articles in the analysis of “ferroptosis in PD” and “pyroptosis in PD,” respectively. Overall, the top 10 most co-cited references in both fields provided a comprehensive overview of ferroptosis and pyroptosis, including their mechanisms, cellular pathways, and disease relevance, forming a strong evidence base that advances the understanding and potentially shifts disease treatment strategies by targeting ferroptosis and pyroptosis.

### 4.2 Research hotspots and frontiers

Keywords are highly condensed and summarized for the themes and core research content of a paper, which can reflect the distribution and development of different research hotspots in a specific field. Through the visualization and analysis of keywords, keyword clustering and time-zone maps, the research hotspots and development frontiers of “ferroptosis in PD” and “pyroptosis in PD” have been identified in this study, which are summarized as follows:

#### 4.2.1 Ferroptosis in PD

Ferroptosis is a novel mode of programmed cell death that is iron-dependent and closely related to lipid peroxidation, involving multiple molecular and metabolic pathways. Iron accumulation is a critical factor in ferroptosis. Increased iron intake, decreased efflux, or decreased storage can lead to iron overload. Excess Fe^2+^ generates large amounts of ROS via the Fenton reaction, exacerbating oxidative damage and providing oxidizing-capable free radicals that hydroxylate intracellular polyunsaturated fatty acids (PUFAs), generating large amounts of lipid hydroperoxides (LOOHs). These damage DNA, lipid membranes, and other biomolecules, which in turn leads to cell death (Jiang et al., 2021). The genesis of lipid peroxides can occur both through iron ion catalysis and enzymatic reactions, the latter involving key enzymes such as acyl-CoA synthetase long-chain family members (ACSLs), lysophosphatidylcholine acyltransferase 3 (LPCAT3), and arachidonate 15-lipoxygenase (ALOX15) (Bouchaoui et al., 2023). These toxic lipid peroxides can be reduced to lipid alcohols (L-OH) by GSH and GPX4, and the system Xc^−^ is a key regulator of GSH synthesis, and solute carrier family 3 member 2 (SLC3A2) and solute carrier family 7 member 11 (SLC7A11) are major components of this transporter. System Xc^−^ and GPX4 are therefore important targets for the regulation of ferroptosis, and when their metabolism is imbalanced, lipid peroxidation is unchecked, leading to ferroptosis (Dar et al., 2023).

In recent years, a series of studies have confirmed that ferroptosis is associated with PD. Significant increases in iron ion levels are often observed in the substantia nigra region of the brain in patients with PD. Furthermore, regulatory proteins involved in iron metabolism, including iron-regulated protein (IRP)/iron response element (IRE) (Lee and Hyun, 2023), transferrin(Tf)/transferrin receptor(TfR), and ceruloplasmin (CP) (Liu L. et al., 2023) are abnormally expressed or dysfunctional in PD patients. In a mouse model of PD, accumulation of L-OOH, a marker of ferroptosis, and a decrease in GPX4 are found to increase the toxicity of dopaminergic neurons, leading to motor dysfunction in mice (Sun et al., 2023). Increased concentrations of malondialdehyde (MDA), a lipid peroxidation byproduct, and 4-hydroxy-2,3-trans-nonenal (4-HNE), the end-product of ferroptosis, were found in an MPTP-induced rat model of PD, with 4-HNE mediating the inflammatory response through the promotion of Lewy bodies formation in PD (Liu et al., 2021). Neuroinflammatory responses are also closely associated with ferroptosis in PD, as evidenced by the excessive activation of microglia in the brains of PD patients (Guo et al., 2021). Iron accumulation causes alterations in microglial transcription, thereby inducing the release of various cytokines and pro-inflammatory substances, such as IL-1β, TNF-α, and IL-6. This process promotes the uptake of iron by neuronal cells, further exacerbating ferroptosis. Recent discoveries have revealed that the regulatory mechanisms of ferroptosis in PD are also closely related to the p53/SLC7A11/GPX4 and Nrf2-related pathways. p53 activation can downregulate Xc^−^ by inhibiting SLC7A11, these actions further reduces the expression of GSH and GPX4, leading to DN loss and motor deficits in the PD mouse model.(Li S. S. et al., 2021). Nrf2 can enhance the resistance of DA to ferroptosis by regulating iron-homeostasis-related proteins (e.g., IRP), heme metabolism, mitochondrial function and by up-regulating the transcription of GPX protein family (Shen et al., 2024). The evidence mentioned above suggests that ferroptosis is one of the key pathological mechanisms of neuronal injury and dysfunction after PD, making it an important target for intervention.

Currently, therapeutic studies targeting ferroptosis in PD have focused on the use of drugs such as iron chelators, antioxidants, and autophagy activators. For instance, iron chelators like deferoxamine have been shown to reduce iron deposition in SN of PD patients, thereby mitigating the progression of motor disorders (Negida et al., 2024). Antioxidants targeting ferroptosis, including N-Acetylcysteine (NAC), Vitamin E, Coenzyme Q10, ACSL4 inhibitors, LOX inhibitors, GPX4 activators, and Nrf2 activators, have also shown potential in slowing PD progression in the study. For example, the probiotic *L. lactis* MG1363-pMG36e-GLP-1 can inhibit ferroptosis in brain tissues of PD mice by activating the Keap1-Nrf2-GPX4 signaling pathway and decreasing the level of oxidative stress, thus improving PD symptoms (Yue et al., 2022). Rapamycin targets ferroptosis in PD by activating the autophagy pathway, reducing the accumulation of iron and attenuating lipid peroxidation, offering various neuroprotective effects on PD (Liu T. et al., 2023). In recent years, the treatment of PD with traditional Chinese medicine (TCM) has attracted more and more attention, both herbal components and acupuncture have shown good potential for anti-PD. Forsythia suspense can enhance antioxidant capacity by activating the Nrf2/GPX4 axis and inhibiting the TLR4 signaling pathway, to modulate ferroptosis-mediated inflammatory response, protect dopamine neurons, and alleviate PD (Zhang et al., 2024). In the 6-OHDA-induced PD rat model, acupuncture treatment at acupoints such as Baihui (GU20) and Taichong (LR3) can promote the release of DA and inhibit the production and aggregation of α-syn in the SN, reducing the loss of dopaminergic neurons and improving the motor symptoms in PD rats (Wang et al., 2024). Moreover, acupuncture therapy can also reduce oxidative stress by activating the Nrf2/ARE pathway (Huang and Hsieh, 2021). Moxibustion at Baihui acupoint can increase the expression of GPX4 in the SN of PD model rats, reduce the level of ROS, improve ferroptosis injury, protect dopaminergic neurons, and thus improve their motor disorders (Huang et al., 2021). The above studies suggest that inhibition of ferroptosis is a potentially important intervention for PD, but more direct clinical evidence remains to be explored.

#### 4.2.2 Pyroptosis in PD

In the progression of PD, pyroptosis is closely associated with the release of inflammatory mediators and the excessive activation of neuroglial cells, which together contribute to the neuroinflammatory state characteristic of the disease. Typically, neuronal pyroptosis is triggered by a confluence of intracellular and extracellular factors, including accumulation of neurotoxins, excessive production of ROS by mitochondria, and disruption of mitochondrial autophagy. These factors collectively activate microglia within the CNS, triggering neuroinflammation and leading to a decrease in dopaminergic neurons and aggregation of α-syn proteins (Pike et al., 2021). Once α-syn is released extracellularly, it further activates microglia and NLRP3 inflammasome through the Toll-like receptor/NF-κB pathway, triggering pyroptosis (Li Y. N. et al., 2021). Caspase-1, recruited during pyroptosis, promotes further aggregation of α-syn to generate Lewy bodies with toxic α-syn protofibrils as the main component, and the formation of Lewy bodies further leads to neuronal death and dopamine loss. In addition, caspase-1 catalyzes the maturation and release of IL-1β and IL-18 and pore formation of GSDMD, further damaging dopamine neurons and promoting the progression of PD (Gao et al., 2020). Significantly elevated levels of IL-1β and IL-18, indicative of pyroptosis, have been found in patients with PD (Zhang et al., 2020). Clinically, biomarkers associated with pyroptosis, such as miR-675-5p, miR-1247-5p, and lncH19, can be used for the early diagnosis of PD and therapeutic and prognostic assessment (Liang et al., 2024). In addition to the NLRP3/caspase-1/GSDMD pathway described above, the onset of pyroptosis in PD is also regulated by the TLR4/TAK1/IRF7 and p38-TFEB pathways. TLR4 is an innate immune receptor that is significantly activated in PD. TLR4 can modulate downstream TAK1 expression to upregulate IRF7, which can mediate DA pyroptosis and promote PD by binding to the GSDMD (Quan et al., 2024). In a PD mouse model, P38 inhibits molecular chaperone-mediated autophagy (CMA) by downregulating TFEB, which further inhibits the degradation of NLRP3 inflammasome (Chen J. L. et al., 2021). These two pathways have the opportunity to be potential therapeutic targets for NLRP3 inflammasome -driven PD.

Activation of the NLRP3 inflammasome-mediated pyroptosis is involved in dopamine neuron death, emerging as a therapeutic target for PD. Numerous NLRP3 inhibitors have been found to intervene in PD by inhibiting the activity of related enzymes and the expression of inflammatory factors. Exendin-4 and linagliptin can be used as novel anti-inflammatory agents in PD by reducing ROS production and attenuating neuroinflammation through inhibition of NLRP3-cellular pyroptosis pathway (Yu et al., 2023). MCC950 attenuates the inflammatory response and reduces murine nigrostriatal inflammatory response through inhibition of NLRP3/IL-1β pathway and reduces hyperphosphorylated α-syn aggregates in the mouse substantia nigra striata thereby protecting dopaminergic neurons and ameliorating PD (Cheng et al., 2020). Transplantation of mesenchymal stem cells and their exosomes has shown great potential in the treatment of PD. Transplantation of adipose mesenchymal stem cell-derived exosomes in the substantia nigra resulted in a significant reduction in the levels of NLRP3 and pro-inflammatory cytokines, and inhibited the pyroptosis of dopamine neuron cells, as well as improved motor function (Li Q. et al., 2021). It has been found that quercetin (QC) prevents neuronal damage by inhibiting the NLRP3 pathway (Lin et al., 2022), but factors such as its low solubility and poor stability limit its effectiveness. Zhang et al. (Zhao et al., 2023) developed a carrier-free nanomedicine (NanoQC), which improves the brain delivery of QC, predominantly accumulating in the SN, reducing the loss of dopamine neurons, thus realizing an efficient and precise therapeutic effect. Consequently, nanotechnology combined with targeted drugs or physical modalities are new and highly promising therapeutic strategies for PD.

#### 4.2.3 The association between ferroptosis and pyroptosis

The above studies highlight the critical roles of ferroptosis and pyroptosis in the development of PD, with both being interconnected at multiple levels. In terms of the inflammasome pathway, excessive iron accumulation can trigger NLRP3 inflammasome activation through the CGAS-STING1 pathway, leading to oxidative stress and ferroptosis in cells (Gupta et al., 2023). In addition, the ferroptosis inhibitor ferrostatin-1 can reduce the expression of NLRP3, IL-1β, and caspase-1, while the ferroptosis inducer erastin can drive the activation of inflammasomes in cells (Li M. H. et al., 2021). Therefore, changes in inflammasome activity also accompany alterations in ferroptosis. As mentioned earlier, the activation of the NLRP3 inflammasome is a crucial triggering event in the neuronal cell pyroptosis pathway. Therefore, it is speculated that pyroptosis and ferroptosis have a synergistic effect in the inflammatory response mediated by the inflammasome pathway. In the MAPK signaling pathway, in a neonatal rat model of hypoxic–ischemic injury, the TLR4-p38MAPK pathway is activated, promoting the production of inflammatory cytokines IL-1β and IL-18, while decreasing the expression of SLC7A11 and GPX4, leading to neuroinflammation and ferroptosis (Zhu et al., 2021). Moreover, iron accumulation and excessive lipid peroxidation have been shown to affect the activity of the MAPK signaling pathway. In mouse models treated with pNaktide, the MAPK signaling pathway is inhibited, resulting in reduced cleavage of GSDMD mediated by caspase-1 and decreased expression levels of pyroptosis-related genes, exerting neuroprotective effects (Zong et al., 2023). This indicates that the MAPK pathway not only plays a role in the inflammatory activation of pyroptosis but also has an important relationship with ferroptosis. In terms of the autophagy-related pathway, autophagy can degrade ferritin, thereby increasing iron levels, ultimately leading to the accumulation of ROS and triggering ferroptosis (Yang et al., 2024). Components of the NLRP3 inflammasome (including NLRP3 and ASC) are co-localized with autophagosomes, which can be phagocytosed and degraded by autophagosomes. Therefore, inhibiting the autophagy pathway can enhance NLRP3 inflammasome activation, thereby mediating pyroptosis (Zhao et al., 2021). Autophagy has opposing effects on pyroptosis and ferroptosis, and exploring the balance point is crucial for disease treatment. The shared pathways of ferroptosis and pyroptosis in PD pathogenesis are currently not well understood. In the future, we can focus on studying the common molecular pathways of pyroptosis and ferroptosis, further clarifying the relationship between neuronal damage in PD patients and pyroptosis and ferroptosis. This will provide new directions for the discovery of specific biomarkers and novel targeted therapeutic drugs for PD patients.

## 5 Limitations

This is the first comprehensive bibliometric analysis of “ferroptosis in PD” and “pyroptosis in PD,” but there are some limitations. First, the search was conducted on February 16, 2024, but the database is continuously updated. Second, the completeness of keyword inclusion in the literature search process may have affected the results due to partial keyword analysis. Third, due to the limitations of the Citespace software, only relevant literature in the WOSCC database was analyzed in this study, and some articles were not included in the WOSCC database and were excluded, resulting in source bias. Finally, the variable quality of the literature selected for this study may reduce the credibility of the overall analysis.

## 6 Conclusion

The fields of “ferroptosis in PD” and “pyroptosis in PD” involve an increasing number of scholars, organizations, and countries, resulting in a significant number of high-quality publications. Particularly, “ferroptosis in PD” has witnessed a rapid increase in research outputs in recent years and will become a research hotspot in the field of PD in the coming period. Furthermore, elucidating the relationship between ferroptosis and pyroptosis may offer new directions for the treatment of PD.

## Data availability statement

The original contributions presented in the study are included in the article/Supplementary material, further inquiries can be directed to the corresponding authors.

## Author contributions

ZW: Investigation, Methodology, Writing – original draft, Writing – review & editing. KZ: Investigation, Methodology, Writing – original draft, Writing – review & editing. BT: Funding acquisition, Writing – review & editing. SX: Writing – review & editing, Investigation.
