# Supplementary material for: Research trends of ferroptosis and pyroptosis in Parkinson’s disease: a bibliometric analysis
Source: Front Mol Neurosci. 2024 May 16;17:1400668. doi: 10.3389/fnmol.2024.1400668 (PMC11137268; doi:10.3389/fnmol.2024.1400668)
Supplement: Supplementary file 1 [file Table_1.DOCX]

**Supplementary Table 1.** Top 10 journals and co-cited journals related to ferroptosis, pyroptosis in Parkinson’s disease

| **Type** | **Ran**  **k** | **Journal** | **Docu**  **ments** | **Cita**  **tions** | **IF**  **(2022)** | **JCR** | **Ran**  **k** | **Co-cited**  **journal** | **Cita**  **tions** | **IF**  **(2021)** | **JCR** |
| --- | --- | --- | --- | --- | --- | --- | --- | --- | --- | --- | --- |
| Ferroptosis  in PD | 1 | Free radical biology  and medicine | 14 | 1308 | 7.4 | Q1 | 1 | Cell | 829 | 64.5 | Q1 |
|  | 2 | International journal  of molecular sciences | 13 | 219 | 5.6 | Q1 | 2 | J Biol Chem | 812 | 4.8 | Q2 |
|  | 3 | Antioxidants | 10 | 79 | 7 | Q1 | 3 | Free Radical Bio Med | 743 | 7.4 | Q1 |
|  | 4 | Oxidative medicine  and cellular longevity | 8 | 338 | 7.31 | Q2 | 4 | P Natl Acad Sci Usa | 702 | 11.1 | Q1 |
|  | 5 | Ageing research reviews | 7 | 146 | 13.1 | Q1 | 5 | Nature | 682 | 64.8 | Q1 |
|  | 6 | Frontiers in neuroscience | 5 | 812 | 4.3 | Q2 | 6 | J Neurochem | 600 | 4.7 | Q2 |
|  | 7 | Frontiers in pharmacology | 5 | 150 | 5.6 | Q1 | 7 | Redox Biol | 488 | 11.4 | Q1 |
|  | 8 | Molecular neurobiology | 5 | 414 | 5.1 | Q2 | 8 | Cell Death Differ | 437 | 12.4 | Q1 |
|  | 9 | Neural regeneration research | 5 | 229 | 6.1 | Q1 | 9 | Plos One | 436 | 3.7 | Q2 |
|  | 10 | Neuroscience | 5 | 112 | 3.3 | Q3 | 10 | J Neurosci | 424 | 5.3 | Q1 |
| Pyroptosis  in PD | 1 | International journal of molecular sciences | 23 | 516 | 5.6 | Q1 | 1 | Nature | 1651 | 64.8 | Q1 |
|  | 2 | Journal of  neuroinflammation | 21 | 563 | 9.3 | Q1 | 2 | P Natl Acad Sci Usa | 1158 | 11.1 | Q1 |
|  | 3 | Frontiers in immunology | 16 | 986 | 7.3 | Q1 | 3 | J Neuroinflamm | 1139 | 9.3 | Q1 |
|  | 4 | Frontiers in pharmacology | 16 | 384 | 5.6 | Q1 | 4 | Cell | 959 | 64.5 | Q1 |
|  | 5 | Antioxidants | 14 | 316 | 7 | Q1 | 5 | Plos One | 922 | 3.7 | Q2 |
|  | 6 | Molecular neurobiology | 14 | 357 | 5.1 | Q2 | 6 | J Biol Chem | 885 | 4.8 | Q2 |
|  | 7 | Frontiers in aging neuroscience | 12 | 149 | 4.8 | Q2 | 7 | J Neurosci | 876 | 5.3 | Q1 |
|  | 8 | International immunopharm-acology | 10 | 472 | 5.6 | Q1 | 8 | Science | 712 | 56.9 | Q1 |
|  | 9 | Neural regeneration research | 9 | 117 | 6.1 | Q1 | 9 | J Immunol | 692 | 4.4 | Q2 |
|  | 10 | Brain behavior and immunity | 8 | 446 | 15.1 | Q1 | 10 | Int J Mol Sci | 689 | 5.6 | Q1 |

**Supplementary Table 2.** Top 10 co-cited references associated with ferroptosis, pyroptosis in Parkinson’s disease

| **Type** | **Ran**  **k** | **Litera**  **ture** | **Cita**  **tions** | **Title** | **DOI** | **Source** |
| --- | --- | --- | --- | --- | --- | --- |
| Ferroptosis in PD | 1 | Dixon Sj  (2012) | 207 | Ferroptosis: an  iron-dependent form of  nonapoptotic cell  death | 10.1016/J.Cell.2012.03.042 | Cell |
|  | 2 | Do Van B (2016) | 135 | Ferroptosis, a  newly characterized form  of cell death in  Parkinson's  disease that is  regulated by PKC | 10.1016/J.Nbd.2016.05.011 | Neurobiol Dis |
|  | 3 | Stockwell Br (2017) | 110 | Ferroptosis: a  regulated cell  death nexus  linking metabolism, redox  biology, and  disease | 10.1016/J.Cell.2017.09.021 | Cell |
|  | 4 | Yang Ws  (2014) | 99 | Regulation of  ferroptotic cancer  cell death by gpx4 | 10.1016/J.Cell.2013.12.010 | Cell |
|  | 5 | Angeli Jpf (2014) | 85 | Inactivation of the  ferroptosis regulator gpx4 triggers acute  renal failure in  mice | 10.1038/Ncb3064 | Nat Cell Biol |
|  | 6 | Doll S  (2017) | 78 | ACSL4 dictates  ferroptosis sensitivity by shaping cellular  lipid composition | 10.1038/Nchembio.2239 | Nat Chem Biol |
|  | 7 | Kagan Ve  (2017) | 77 | Oxidized arachidonic and  adrenic PEs navigate cells to  ferroptosis | 10.1038/Nchembio.2238 | Nat Chem Biol |
|  | 8 | Hambright Ws  (2017) | 72 | Ablation of  ferroptosis regulator glutathione peroxidase 4 in  orebrain | 10.1016/J.Redox.2017.01.021 | Redox Biol |
|  | 9 | Guiney Sj  (2017) | 70 | Ferroptosis and  cell death  mechanisms in  Parkinson's disease | 10.1016/J.Neuint.2017.01.004 | Neurochem Int |
|  | 10 | Devos D  (2014) | 63 | Targeting chelatable iron as a therapeutic modality in Parkinson's disease | 10.1089/Ars.2013.5593 | Antioxid Redox Sign |
| Pyroptosis  in PD | 1 | Gordon R (2018) | 145 | Inflammasome inhibition prevents  α-synuclein  pathology and  dopaminergic neurodegeneration in mice | 10.1126/Scitranslmed.Aah4066 | Sci Transl Med |
|  | 2 | Heneka Mt (2013) | 142 | NLRP3 is  activated in  Alzheimer's disease and contributes to  pathology in  APP/PS1 mice | 10.1038/Nature11729 | Nature |
|  | 3 | Codolo G (2013) | 133 | Triggering of  inflammasome by  aggregated  α-synuclein, an  inflammatory response in  synucleinopathies | 10.1371/Journal.Pone.0055375 | Plos One |
|  | 4 | Lee E (2019) | 109 | MPTP-driven NLRP3 inflammasome activation in microglia plays  a central role in  dopaminergic  neurodegeneration | 10.1038/S41418-018-0124-5 | Cell Death  Differ |
|  | 5 | Yan Yq  (2015) | 96 | Dopamine controls  systemic inflammation through inhibition  of NLRP3  inflammasome | 10.1016/J.Cell.2014.11.047 | Cell |
|  | 6 | Halle A  (2008) | 93 | The NALP3  inflammasome is  involved  in the innate  immune response  to amyloid-β | 10.1038/Ni.1636 | Nat Immunol |
|  | 7 | Zhou Y  (2016) | 89 | MicroRNA-7 targets nod-like  receptor protein 3  inflammasome to  modulate neuroinflammation in the pathogenesis of Parkinson's  disease | 10.1186/S13024-016-0094-3 | Mol Neurodegener |
|  | 8 | Zhou Rb  (2011) | 85 | A role for  mitochondria in  NLRP3 inflammasome activation | 10.1038/Nature09663 | Nature |
|  | 9 | Mao Zj  (2017) | 68 | The NLRP3  inflammasome is  involved in the  pathogenesis of  Parkinson's disease in rats | 10.1007/S11064-017-2185-0 | Neurochem Res |
|  | 10 | Martinon F  (2002) | 67 | The inflammasome: a  molecular platform  triggering activation of  inflammatory  caspases and  processing of  proIL-β | 10.1016/S1097-2765(02)00599-3 | Mol Cell |
